# Serum HDL-C levels in children with epilepsy: a single-center retrospective study

**DOI:** 10.3389/fnut.2025.1523426

**Published:** 2025-02-20

**Authors:** Hong-Li Guo, Na Dong, Ya-Hui Hu, Jin-Chun Qiu, Zhen-Zhou Jiang, Qian-Qi Liu, Xiao-Peng Lu, Feng Chen

**Affiliations:** ^1^Pharmaceutical Sciences Research Center, Department of Pharmacy, Children’s Hospital of Nanjing Medical University, Nanjing, China; ^2^Institute of Pharmaceutical Sciences, China Pharmaceutical University, Nanjing, China; ^3^Department of Children Health Care, Children’s Hospital of Nanjing Medical University, Nanjing, China; ^4^Department of Neurology, Children's Hospital of Nanjing Medical University, Nanjing, China

**Keywords:** children, epilepsy, anti-seizure medications, high-density lipoprotein cholesterol, vitamin D supplementation

## Abstract

**Purpose:**

This study aims to compare the difference in serum high-density lipoprotein cholesterol (HDL-C) levels between children with epilepsy and healthy children and to assess its potential influencing factors.

**Methods:**

For comparison, we retrospectively collected data on 1,002 children with epilepsy who visited the Department of Neurology at the Children’s Hospital of Nanjing Medical University. Additionally, we included 127 healthy children who underwent routine health examinations at our hospital’s Health Examination Center. This study also incorporated 98 recently diagnosed epilepsy patients who had not yet received treatment with anti-seizure medications (ASMs) as a source of baseline data. Demographic information and laboratory test results were retrieved from the hospital information system. The Kolmogorov–Smirnov test, the Mann–Whitney test, the Fisher’s exact test, odds ratios (OR), Spearman or Pearson correlation coefficients, and *post-hoc* analysis were used to conduct statistical analysis.

**Results:**

Healthy children exhibited significantly higher serum levels of HDL-C compared to children with epilepsy and the baseline values. Notably, a higher percentage of children with epilepsy exhibited a low HDL-C levels (<1.0 mmol/L) compared to healthy children, showing an increased risk of dyslipidemia (OR, 2.773; 95% CI, 0.9879–7.457). The type of ASMs had a notable effect on serum HDL-C levels, particularly with hepatic enzyme-inducing ASMs like oxcarbazepine, which significantly raised the serum HDL-C levels. The serum HDL-C levels were also associated with factors such as age, epilepsy history, and brain magnetic resonance imaging findings. Additionally, there was a weak negative association between serum vitamin D levels and serum HDL-C levels (R = -0.37, *p* = 0.0014). Moreover, children who received vitamin D supplementation demonstrated a higher level of HDL-C than those without such supplementation.

**Conclusion:**

Serum HDL-C levels are notably lower in children with epilepsy than in healthy children. Treatment with ASMs can partially increase the serum HDL-C levels, potentially approaching those found in healthy children. Therefore, the decrease in serum HDL-C levels in children with epilepsy irrespective of receiving ASMs treatment should warrant ongoing attention.

## Highlights

- The serum HDL-C concentrations in children with epilepsy are still not well defined, nor is it clear how ASMs treatment affect HDL-C levels in their serum.
- Children with epilepsy, including those who have recently been diagnosed and have yet to begin ASMs treatment, have significantly lower serum HDL-C levels compared to their healthy peers.
- Treatment with ASMs resulted in a significant increase in serum HDL-C levels, with oxcarbazepine monotherapy raising these levels to those comparable to healthy individuals.
- Supplementing with vitamin D appears to be associated with an increase in serum HDL-C levels.
- The lower serum HDL-C levels in children with epilepsy compared to healthy children, along with their potential association with cardiovascular disease, should raise widespread concern.

## 1 Introduction

In recent years, a large number of studies on the relationship between lipid metabolism and epilepsy have enhanced our understanding of the mechanisms underlying the occurrence and development of epilepsy, as well as identified potential therapeutic targets in lipid pathways (1–3). However, there has been ongoing concern regarding the possible changes in blood lipids in patients with epilepsy. This is because pharmacological treatment of epilepsy requires the use of various anti-seizure medications (ASMs) with different mechanisms, often necessitating several years of treatment (4, 5). Consequently, the potential adverse effects of long-term drug exposure, such as impacts on blood lipids (6), have received significant attention, given that dyslipidemia is closely associated with cardiovascular diseases (CVD) (7, 8).

The current issue is that the impact of ASMs on blood lipids remains inconclusive. One particular point of concern is that there are few reports on the blood lipid levels of patients who have just been diagnosed with epilepsy but have not yet started any medication. Additionally, there is a lack of studies that include healthy individuals as controls, which can significantly affect the assessment of changes in blood lipids.

Indeed, the impact of ASMs therapy on serum high-density lipoprotein cholesterol (HDL-C) levels and its relationship with atherosclerosis is a topic of debate. Research has produced conflicting findings regarding the long-term effects of ASMs therapy on blood lipid levels, with some studies indicating significant effects while others did not (9–11). Some studies revealed that patients with epilepsy receiving ASMs therapy have a reduced risk of heart disease associated with atherosclerosis (12, 13), whereas other studies report a slight increase in mortality rates due to heart disease (14).

For children with epilepsy, there is also a focus on changes in vitamins, such as vitamin D, which is crucial for the normal development of both the skeletal and non-skeletal systems in children (15). Intriguingly, studies revealed that the unfavorable lipid profile found in people with vitamin D deficiency (16), but vitamin D supplementation led to notable improvements in lipid profiles, including increased HDL-C levels and decreased triglycerides (TG) levels (17, 18). Indeed, while some studies have found a notable association between serum HDL-C levels and the circulating concentration of 25-OH-VitD (a major marker for assessing vitamin D status), others have not observed such a connection in children (19). However, the association between serum HDL-C levels and vitamin D status in children with epilepsy have not yet been investigated.

In this research, we aimed to compare the difference in serum HDL-C levels between children with epilepsy and healthy controls, and to assess its potential influencing factors, such as ASMs therapy and vitamin D status, in children with epilepsy.

## 2 Materials and methods

### 2.1 Study population

This case–control study encompassed children (0–18 years of age) with epilepsy, as well as a group of healthy controls. The study had a retrospective design covering children with epilepsy included from February 11, 2019 to June 27, 2022. They performed routine blood biochemistry panel, which included various parameters such as serum HDL-C levels as a minimum requirement attending in the Department of Neurology, Children’s Hospital of Nanjing Medical University. For impacting factors on the serum HDL-C status analysis, children with epilepsy were excluded who were not treated with ASMs or lacked 25-OH-VitD data.

In addition, we also incorporated newly diagnosed epilepsy children, who had not yet initiated ASMs therapy and thus served as the baseline in the same hospital from January 14, 2019 to June 27, 2022. Those children without serum HDL-C level tests were excluded.

Healthy children who did regular physical examination, were investigated as controls, with serum HDL-C levels measured from June 4, 2022 to July 16, 2022, at our hospital’s physical examination center of the Department of Child Health Care. Those children with diagnosis with congenital heart disease, pygmyism, hypoevolutism, obesity, tie disorder, attention deficithy peractivity disorder, and autism were excluded.

**Figure 1:**
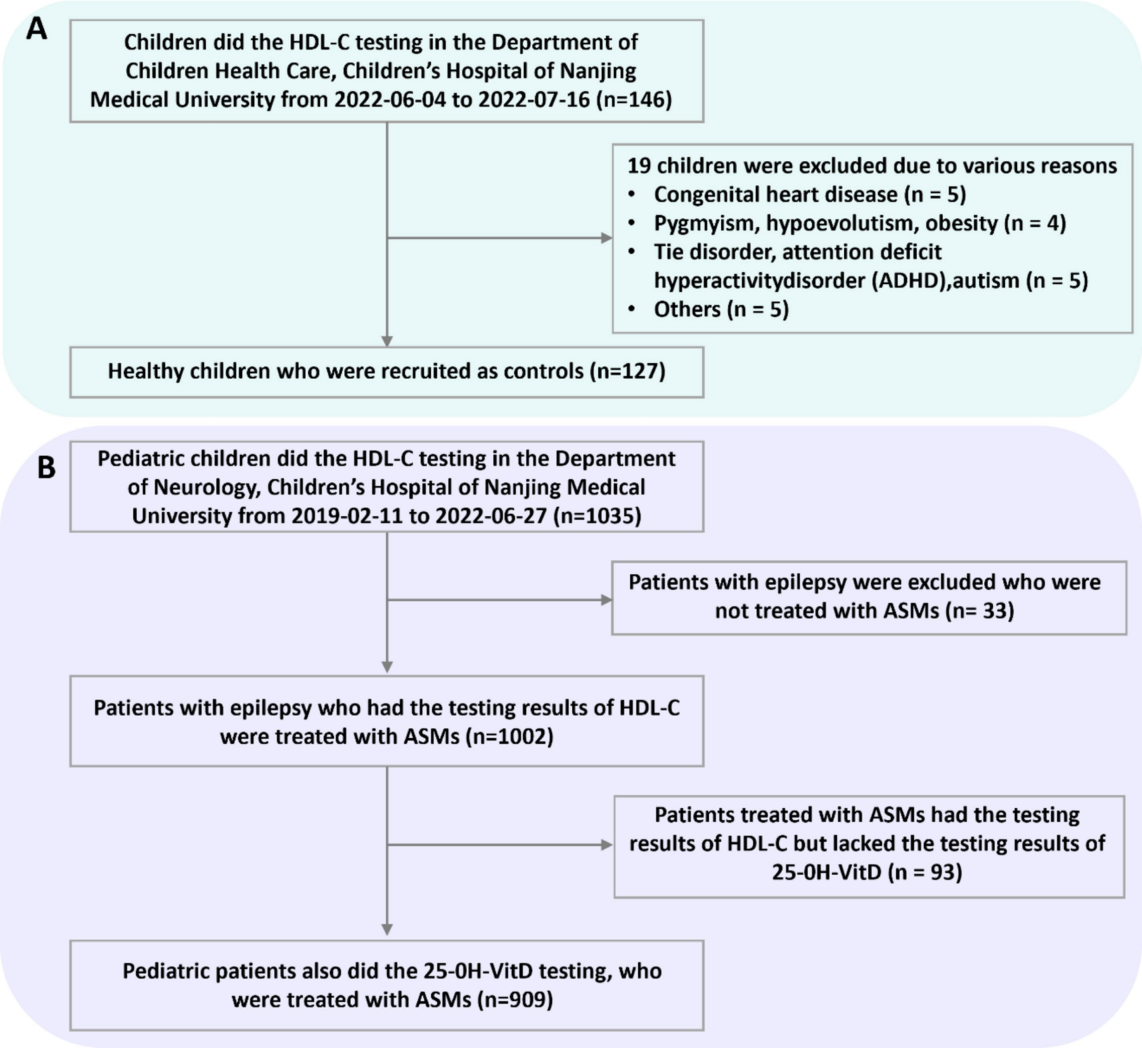
Numbers of healthy children **(A)** and children with epilepsy **(B)** who were eligible for the study.

Figure 1 illustrates the inclusion and exclusion criteria. In the present retrospective study, a convenience sampling approach was employed. This entailed that all cases fulfilling the inclusion and exclusion criteria were incorporated into the study, without implementing strict matching procedures between the two groups during the enrollment phase.

### 2.2 Definitions

According to the reference standards for dyslipidemia in children and adolescents (20, 21), the serum HDL-C status was considered to be acceptable (≥1.20 mmol/L), borderline-high (1.0–1.2 mmol/L), and low (<1.0 mmol/L), respectively.

According to the guidelines of the Endocrine Society of America (22), the serum vitamin D status was classified as sufficient (serum 25-OH-VitD >50 nmol/L), insufficient (serum 25-OH-VitD 30–<50 nmol/L), and deficient (serum 25-OH-VitD <30 nmol/L), respectively.

Furthermore, we categorized childhood into 4 different stages, namely infancy (28 to 364 days old), early childhood (1–6 years of age), middle childhood (6 to 12 years old), and adolescence (12–18 years of age), respectively (23).

The ASMs were grouped and analyzed based on their classification as enzyme-inducing-ASMs (EIASMs) or non-enzyme-inducing-ASMs (NEIASMs). The EIASMs group included children who utilized one or multiple EIASMs, regardless of their utilization of NEIASMs.

The term “seizure-free” was used to describe patients who showed no signs of seizures while continuing to take their prescribed medications. On the other hand, the term “seizure” referred to patients who experienced less than a 50% decrease in seizure frequency while keeping their medications unchanged.

### 2.3 Clinical data collection

A standard proforma sheet was used to gather clinical data from the hospital information system. This proforma sheet contained a range of data, including the following:

1. The demographic data of potential factors that might influence the serum HDL-C status, like age, body weight, and sex, were collected. Additionally, children should maintain a normal diet 3 days prior to blood collection. Fasting blood collection was required on the day of sampling. The following points require further clarification:
  Fasting blood collection was defined as the collection of venous blood in the early morning before eating and at least 8 to 14 h after the last meal. However, for infants and young children, it was necessary to ensure the quality of blood sample collection while not fasting them for too long. The fasting time could be controlled according to the following rules: breastfed children only need to fast for 2 to 3 h; formula-fed children only need to fast for 3 to 4 h; for children who had started complementary foods, such as eating noodles or porridge, generally fast for 5 to 6 h. For children who eat the same diet as adults, such as rice and meat, they need to fast for at least 8 h.
2. Epilepsy history: the outcomes reported by the patient, their response to antiseizure therapy, the medications they took EIASMs like oxcarbazepine (OXC), phenytoin (PHT), perampanel (PER), and topiramate (TPM) at a dosage of ≥200 mg/day; and NEIASMs such as clonazepam (CZP), lacosamide (LAC), lamotrigine (LTG), levetiracetam (LEV), TPM at a dosage of <200 mg/day, vigabatrin (VGB), and VPA, the number of ASMs prescribed, duration and dosage of the drugs used, and the data on the concentration monitoring of the ASMs.
3. Vitamin D supplementation and serum 25-OH-VitD levels. In the routine clinical practice for vitamin D supplementation, children with epilepsy who had vitamin D insufficiency or deficiency were being given a 700 IU of vitamin D_3_ (according to drug specifications) for a minimum duration of 3 months. After 3 months, the levels of vitamin D were measured. If vitamin D levels did not increase to a sufficient status, supplementation would be continued until adequate levels were restored. At that time, the supplementation would be discontinued.
4. Brain magnetic resonance imaging (MRI) scans and their observations.
5. Additional demographic and clinical data retrieved from the medical records of each patient.
6. If there were measurements taken over time for the same patient, who may also have changes in all other variables over time (age, body weight, vitamin D dosage, and medication use, etc.), then this was also collected as a new record.

### 2.4 Statistical analysis

We conducted the statistical analysis using SPSS version 26.0 software (IBM, Armonk, United States) and GraphPad Prism 9.0 (GraphPad Software, La Jolla, CA, United States) with a two-tailed significance levels set at *p* < 0.05. Normality was assessed using Shapiro–Wilk tests. Categorical variables were expressed as numbers (n) and percentages (%), while continuous variables were reported as median and interquartile range. The Kolmogorov–Smirnov test was utilized to identify differences among the groups. For comparing quantitative data between two groups, the nonparametric Mann–Whitney test was employed. The Fisher’s exact test was used to analyze and compare nominal data in the three groups. Odds ratios (ORs) with 95% confidence interval (CI) were calculated between case and control group. *Post-hoc* analysis was employed to evaluate whether the sample size utilized was adequate to detect the actually observed effect size. Depending on the distribution of the variables, either Spearman or Pearson correlation coefficients were used to conduct correlation analyses of potential factors that may affect HDL-C levels. Multivariate linear regression analyses were performed to further discern potential factors influencing the HDL-C levels.

### 2.5 Legal and ethical considerations

The study was conducted in compliance with the guidelines of the Helsinki Declaration. Approval for the collection of medical data was granted by the Ethics Committee of the Children’s Hospital of Nanjing Medical University (Protocol number 202306008-1). Due to the retrospective design of the study, informed consent from parents or guardians was not required.

## 3 Results

### 3.1 Characteristics of the enrolled pediatric subjects

In this study, 127 controls and 1,002 cases with 1884 tests were finally included for the retrospective analyses on the serum HDL-C levels, respectively (Figure 1). A total of 98 patients, who were children with epilepsy and had not received any treatment (baseline group), participated in the current study. The *post hoc* power value between the epilepsy group (*n* = 1884) and the healthy group (*n* = 127) was 0.99 while the power between the baseline group (*n* = 98) and the healthy group (*n* = 127) was only 0.43. Table 1 presents the primary features of the study population. The median ages of children in the healthy, baseline, and epilepsy groups were 7, 5, and 6 years old, respectively. In terms of age, sex, and body weight, the healthy group and the epilepsy group exhibited similarities. However, the median age in the healthy group was significantly higher than that in the baseline group. The proportions of male were 62.2, 57.14, and 54.59% among the three group, respectively.

**Table 1:** Demographic characteristics of healthy children and pediatric patients with epilepsy.

Characteristic	Healthy children	Baseline	Children with epilepsy
N	127	98	1,002
Records of serum HDL-C testing	127	98	1884
Age (years)			
Median (interquartile range)	7 (5–9)	5 (3–7)	6 (3–9)
Growth Stage (*n*, %)			
Infancy (29–364 days)	1 (0.79)	5 (5.10)	80 (4.25)
Early Childhood (1–6 years)	42 (33.07)	64 (65.31)	753 (39.97)
Middle Childhood (6–12 years)	69 (54.33)	22 (22.44)	820 (43.52)
Adolescence (12–18 years)	15 (11.81)	7 (7.14)	231 (12.26)
Sex (*n*, %)			
Male	79 (62.20)	56 (57.14)	547 (54.59)
Female	48 (37.80)	42 (42.86)	455 (45.41)
HDL-C (mmol/L)			
Median (interquartile range)	1.58 (1.33–1.85)	1.32 (1.13–1.59)	1.46 (1.25–1.76)

### 3.2 HDL-C status in healthy children and children with epilepsy

**Figure 2:**
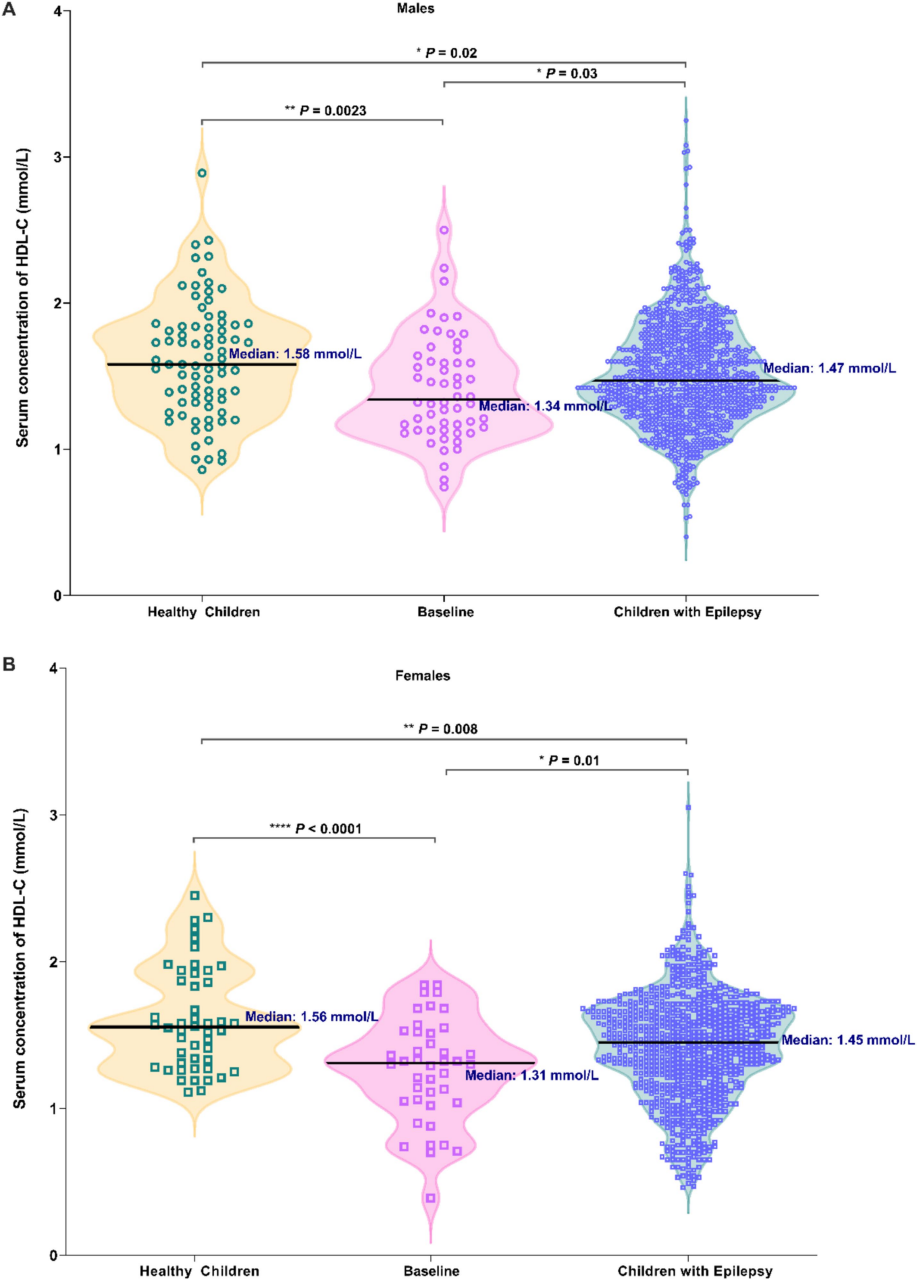
Comparison in the serum HDL-C levels among three groups of individuals: healthy children, newly diagnosed epileptic children who have not received ASM treatment (Baseline), and children with epilepsy (Males: **A**; Females: **B**).

As shown in Figure 2, significantly higher serum HDL-C levels in male healthy group were found when compared with another two arms (*p* = 0.0023, *p* = 0.02). A notable decrease was observed in HDL-C levels among males with epilepsy prior to ASMs therapy (*p* = 0.03, Figure 2A). Similar findings were also found in female cases (*p* < 0.0001, *p* = 0.008, *p* = 0.01, Figure 2B), with these differences being particularly accentuated in females.

In the baseline group and the epilepsy group, the proportions of dyslipidemia (characterized by HDL-C levels <1 mmol/L) were relatively elevated compared to those in the healthy group, yet the differences did not reach statistical significance (Table 2). However, the proportions of acceptable HDL-C levels (characterized by HDL-C levels ≥1.2 mmol/L) in baseline group and epilepsy group were significantly lower than the healthy group (Table 2, *p* = 0.0047). Furthermore, when compared with healthy children, patients with epilepsy exhibited an increased risk of dyslipidemia (OR, 2.773; 95% CI, 0.9879–7.457, Table 3). Similarly, the baseline group also manifested an elevated risk of dyslipidemia in contrast to healthy children (OR, 2.126; 95% CI, 0.8895–4.922, Table 3).

**Table 2:** Proportion of acceptable, brderline-high, and low plasma HDL-C levels in healthy, baseline, and epilepsy groups.

HDL-C status (mmol/L)	Epilepsy (*n*, %)	Baseline (*n*, %)	Healthy (*n*, %)	*p*^a^	*p*^b^
Acceptable (≥1.2)	1,473 (78.2)	64 (65.3)	106 (83.5)	0.1576	0.0047
Borderline-high (1.0–1.2)	260 (13.8)	24 (24.5)	16 (12.6)		
Low (<1.0)	151 (8.0)	10 (10.2)	5 (3.9)		

a Fisher’s exact test to compare the proportion differences among the three groups between <1.0 and ≥1.0 mmol/L.

b Fisher’s exact test to compare the proportion differences among the three groups between <1.2 and ≥1.2 mmol/L.

**Table 3:** The risk of dyslipidemia in children with epilepsy.

HDL-C status (mmol/L)	Epilepsy (*n*)	Baseline (*n*)	Healthy (*n*)	OR (95%CI)^a^	OR (95%CI)^b^
≥1.0	1733	88	122	2.773 (0.9879–7.457)	2.126 (0.8895–4.922)
<1.0	151	10	5		

OR, odds ratio.

a Epilepsy group vs. Healthy group.

b Baseline group vs. Healthy group.

### 3.3 Impacting factors on the serum HDL-C status

#### 3.3.1 Age

**Figure 3:**
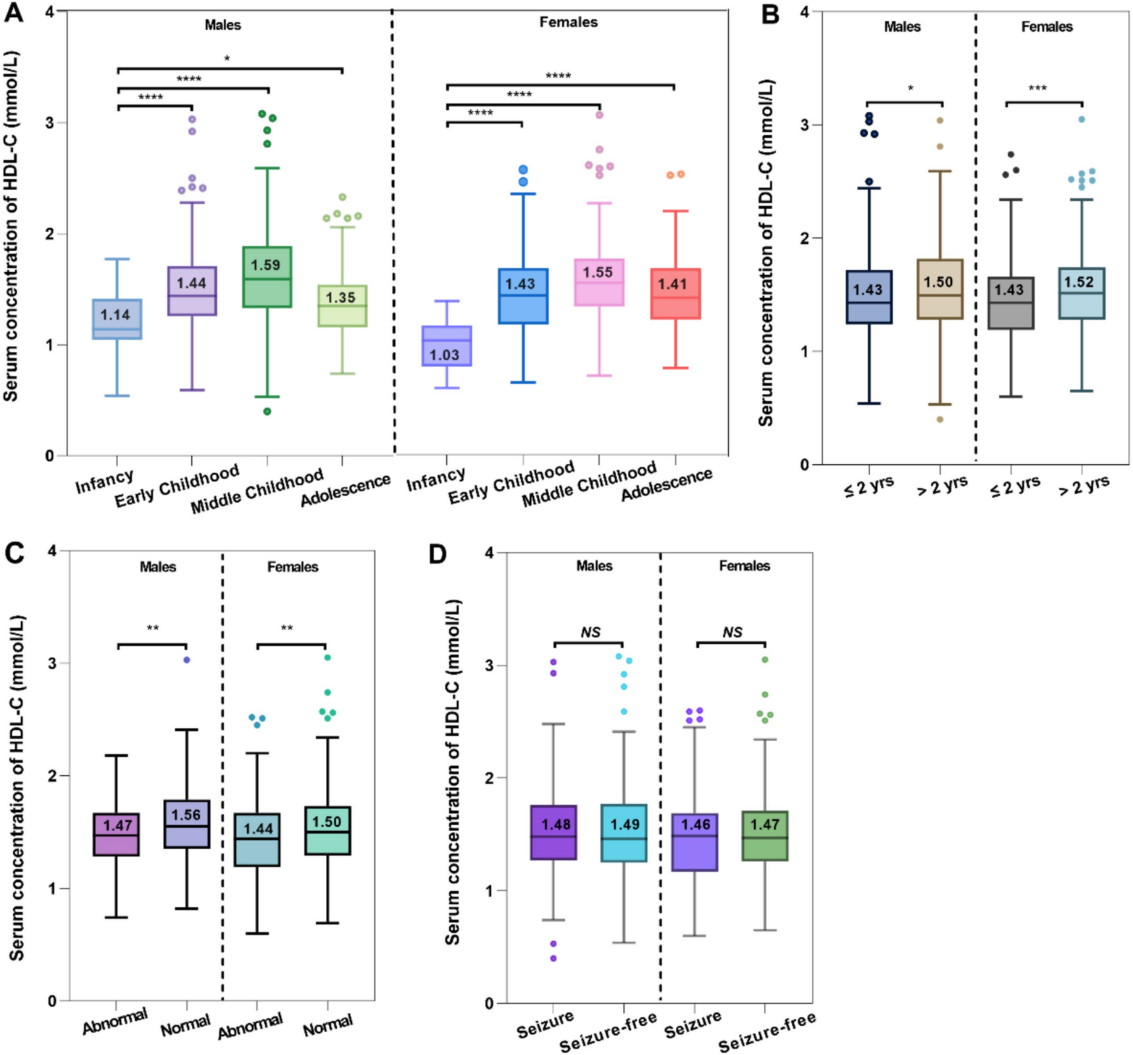
Comparative analysis between the serum HDL-C levels and age **(A)**, epilepsy history **(B)**, MRI findings **(C)**, and seizure control **(D)**.

The serum HDL-C levels displayed a consistent upward pattern from infancy to middle childhood, irrespective of sex. Of note, the male and female children experienced approximately a 1.4- and 1.5-times increase in median serum HDL-C levels from infancy to middle childhood, respectively (Figure 3A). It was important to highlight that children with epilepsy in infancy had significantly lower serum HDL-C levels compared to those in the other three age stages.

#### 3.3.2 Epilepsy history

There were notable disparities in epilepsy history regarding the serum HDL-C levels occurred in both male and female pediatric cases (Figure 3B). Compared to individuals with longer durations of epilepsy (>2 years, HDL-C median = 1.50 mmol/L for males, and HDL-C median = 1.52 mmol/L for females), those with shorter durations (≤2 years, HDL-C median = 1.43 mmol/L for males, and HDL-C median = 1.43 mmol/L for females) experienced a more pronounced negative impact on their serum HDL-C levels. Intriguingly, the rise in serum HDL-C levels was more noticeable among female patients who had experienced epilepsy for over 2 years.

#### 3.3.3 Brain MRI scan results

288 children with epilepsy underwent MRI scans, during which their serum 25-OH-VitD and HDL-C levels were assessed. Among the 153 pediatric patients who had normal MRI scans, it was discovered that their serum HDL-C levels were considerably higher compared to the children (*n* = 135) who had abnormal findings (males: median 1.56 vs. 1.47 mmol/L, *p* = 0.003; females: median 1.44 vs. 1.50 mmol/L, *p* = 0.008; Figure 3C).

#### 3.3.4 ASMs treatment response

No discernible variation in serum HDL-C levels was observed between children with epilepsy who had seizures and those who were seizure-free (Figure 3D).

#### 3.3.5 ASMs for epilepsy therapy

**Figure 4:**
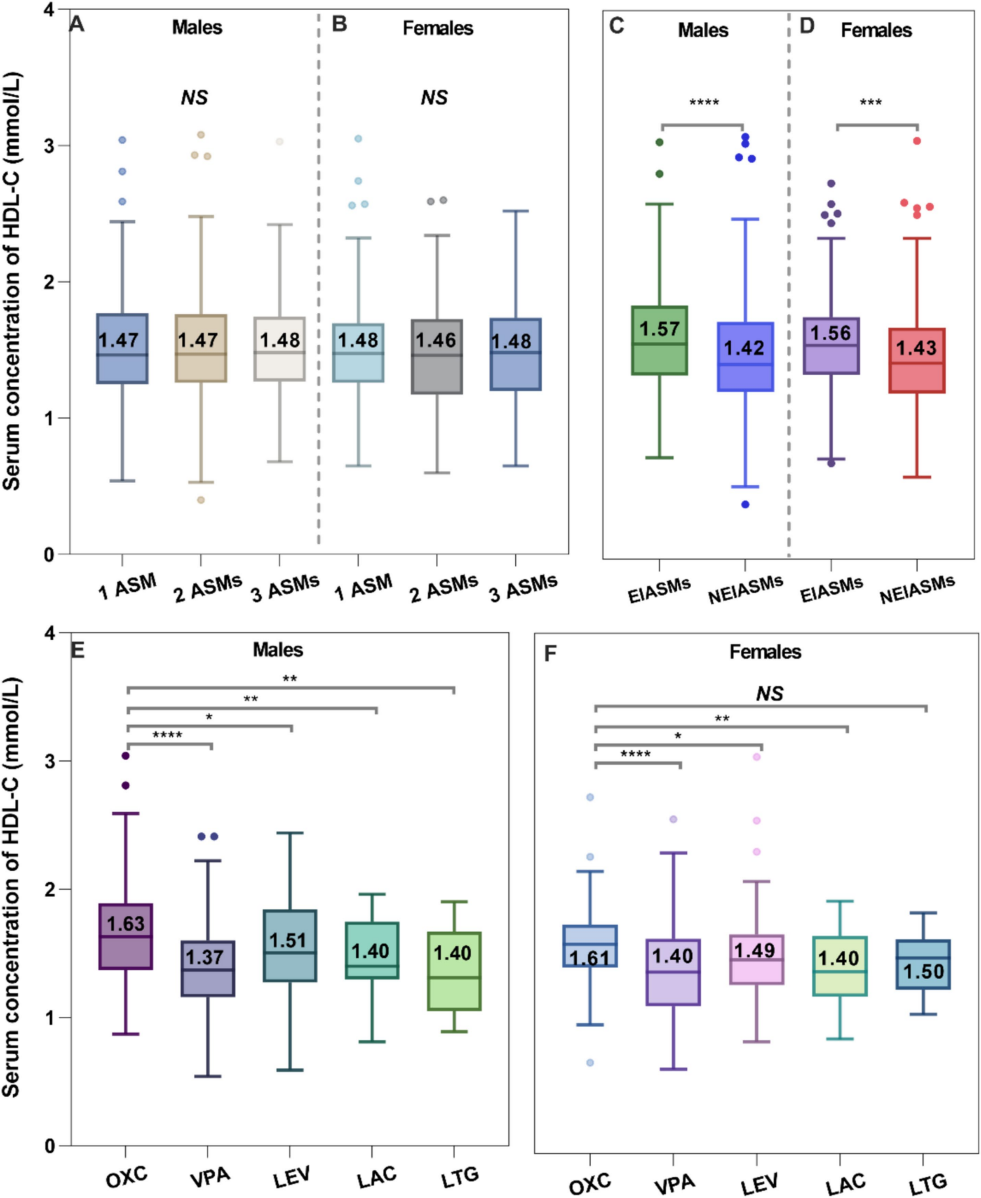
Effects of the number of ASMs taken **(A,B)**, type of ASMs **(C,D)**, and ASM monotherapy on the serum HDL-C levels **(E,F)**.

Firstly, number of administered ASMs did not show any correlation with serum HDL-C levels (Figure 4A). However, the type of ASMs utilized was found to be associated with changes in serum HDL-C levels. A substantial variation in serum HDL-C levels was observed when comparing the EIASMs and NEIASMs group. Without inducers, NEIASMs led to a significant decrease in the HDL-C levels regardless of the sex of children with epilepsy (*p* < 0.0001, *p* = 0.0002, Figure 4B). In addition, OXC taken alone was associated with significantly increased lipid levels. Pediatric patients with epilepsy who were administrated OXC regimens showed significantly higher serum HDL-C levels than those receiving any other monotherapy (Figure 4C). During the course of therapy, no correlation was observed between alterations in serum HDL-C levels and plasma ASMs concentrations (data not shown).

#### 3.3.6 Relationship between serum vitamin D level and serum HDL-C status

A total of 909 individuals (490 males and 419 females) were classified to be suitable for analyzing the impact of serum 25-OH-VitD status on serum HDL-C concentrations in children with epilepsy. And 68 untreated patients (37 males and 31 females) were also identified to analyze the association between vitamin D level and HDL-C status. Characteristics of these study subjects were present in Table 4. Sample size power analysis for the vitamin D-HDL correlation was carried out in the baseline and epilepsy groups. The power was 0.89 in the baseline group and 0.86 in the epilepsy group, respectively.

**Table 4:** Demographic characteristics of pediatric patients with epilepsy, who did both the HDL-C and 25-OH-VitD testing.

Characteristic	Male	Female
*N*	490	419
Records of serum HDL-C and 25-OH-VitD testing	901	742
HDL-C (mmol/L)		
Median (interquartile range)	1.47 (1.26–1.76)	1.47 (1.24–1.71)
25-OH-VitD (nmol/L)		
Median (interquartile range)	51.54 (41.17–66.31)	49.70 (38.26–65.97)
Age (years)		
Median (interquartile range)	6 (4–9)	6 (3–9)
Growth Stage (*n*, %)		
Infancy (29–364 days)	28 (3.11)	22 (2.97)
Early Childhood (1–6 years)	350 (38.84)	308 (41.51)
Middle Childhood (6–12 years)	395 (43.84)	328 (44.20)
Adolescence (12–18 years)	128 (14.21)	84 (11.32)
Body Weight (kg)		
Median (interquartile range)	25 (17–35)	22 (16–35)
Epilepsy history (years)		
Median (interquartile range)	2.08 (1–3.67)	2 (0.92–3.67)
MRI findings (n, %)	157	131
Abnormal	82 (52.23)	53 (40.46)
Normal	75 (47.77)	78 (59.54)
Anti-seizure Treatment (*n*, %)		
Number of ASMs		
1 ASM	464 (53.27)	428 (59.69)
2 ASMs	266 (30.54)	188 (26.22)
≥ 3 ASMs	141 (16.19)	101 (14.09)
Type of ASMs		
CZP	57	43
LAC	127	91
LEV	312	294
LTG	140	85
OXC	207	161
PER	50	36
TPM	70	36
VPA	487	359

**Figure 5:**
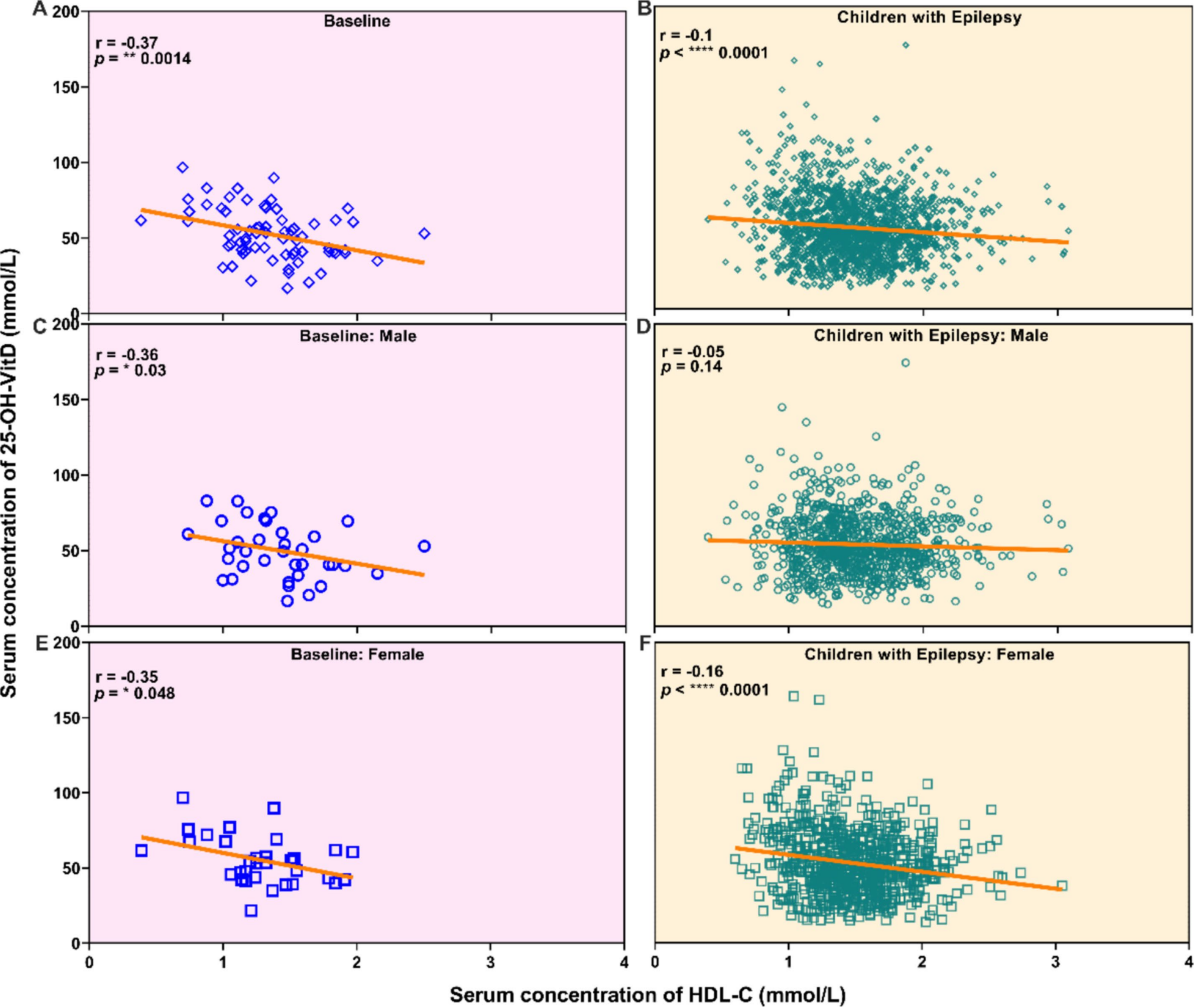
Correlation analysis between the serum vitamin D levels and the serum HDL-C levels in newly diagnosed epileptic children who have not received ASM treatment (All: **A**, Males: **C**, Females: **E**), and children with epilepsy (All: **B**, Males: **D**, Females: **F**).

**Figure 6:**
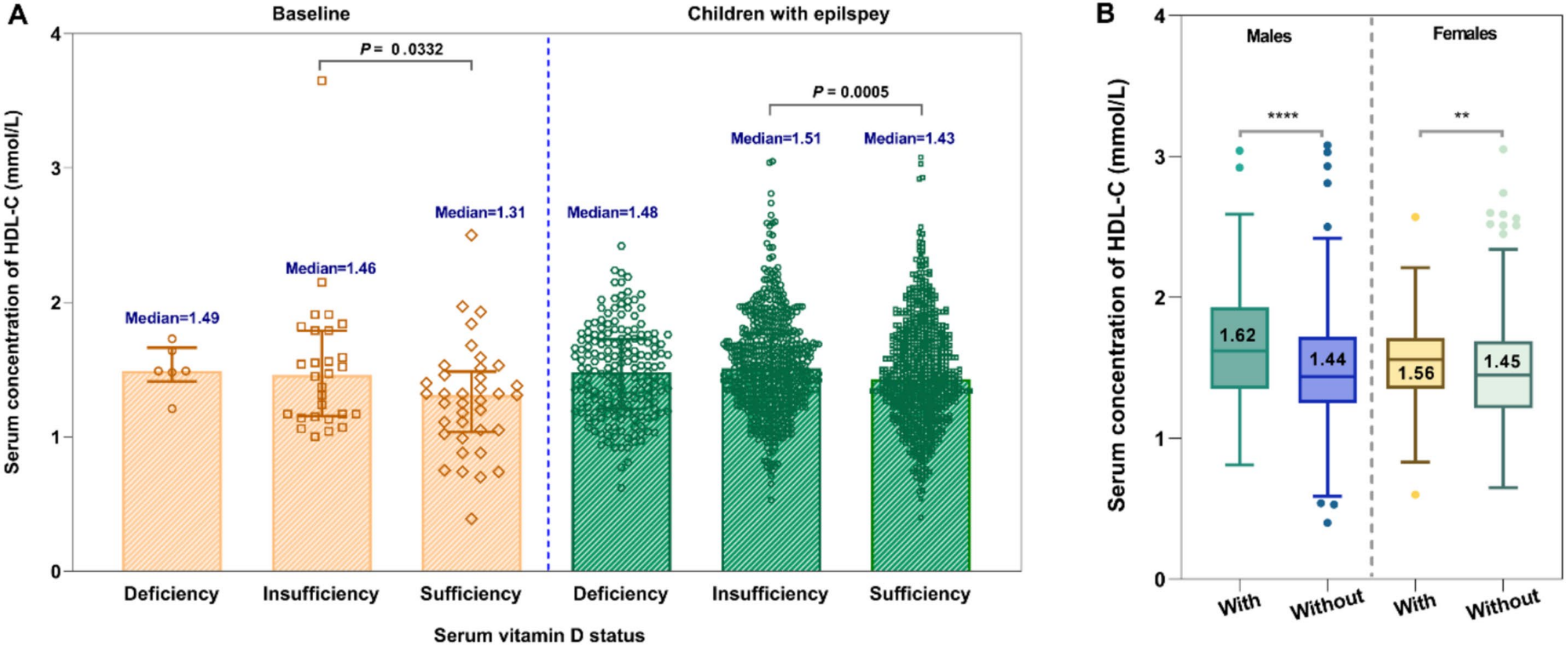
Comparative analysis of the serum HDL-C levels in children under different serum vitamin D status **(A)** and the effect of vitamin D supplementation on HDL-C levels **(B)**.

For untreated group, there was a slight negative association between vitamin D levels and HDL-C levels (Figures 5A,C,E). However, no significant correlation was observed between serum 25-OH-VitD status and plasma HDL-C levels among these 909 pediatric patients (Figures 5B,D,F). Both in the baseline group and the epilepsy group, it was shown that children with insufficient levels of vitamin D had higher HDL-C levels (*p* = 0.0332, *p* = 0.0005, Figure 6A). Interestingly, the serum HDL-C levels of patients who received vitamin D supplementation were significantly higher than those of patients who did not (*p* = 0.009, Figure 6B).

#### 3.3.7 Multivariate linear regression analysis of potential influencing factors

As shown in Table 5, in males, multivariate linear regression analysis indicated higher serum HDL-C levels in early and middle childhood compared to infancy (*β* = 0.2504, *p* = 0.0005; β = 0.3301, *p* < 0.0001). NEIASMs therapy led to a significant decrease in the HDL-C levels compared to EIASMs therapy (β = −0.0901, *p* = 0.0081). Additionally, children without vitamin D supplementation exhibited lower serum HDL-C levels (β = −0.1411, p < 0.0001).

**Table 5:** Results of multivariate linear regressions in the epilepsy group.

			Serum HDL-C levels (mmol/L)		
Covariates			β	95% CI	*p*-value
Males	Duration of Epilepsy	≤ 2 years	−0.0431	−0.0948 ~ 0.0086	0.1019
	Growth Stage	Early childhood	0.2504	0.1095 ~ 0.3913	0.0005
		Middle childhood	0.3301	0.1852 ~ 0.4751	<0.0001
		Adolescence	0.114	−0.0453 ~ 0.2732	0.1605
	Comedication				
		OXC-mono	0.0665	−0.03 ~ 0.163	0.1766
		NEIASMs	−0.0901	−0.1568 ~ −0.0235	0.0081
	MRI	Normal	0.0088	−0.0737 ~ 0.0914	0.8333
	25-OH-VitD levels				
		Insufficiency	0.0496	−0.0442 ~ 0.1433	0.2996
		Sufficiency	0.009	−0.0865 ~ 0.1045	0.8537
		No supplementation	−0.1411	−0.2056 ~ −0.0766	<0.0001
Females	Duration of Epilepsy	≤ 2 years	−0.0606	−0.1156 ~ −0.0057	0.0307
	Growth Stage	Early childhood	0.4298	0.2784 ~ 0.5812	<0.0001
		Middle childhood	0.5049	0.348 ~ 0.6619	<0.0001
		Adolescence	0.4326	0.255 ~ 0.6102	<0.0001
	Comedication				
		OXC-mono	−0.0892	−0.2033 ~ 0.0249	0.1253
		NEIASMs	−0.1703	−0.2499 ~ −0.0906	<0.0001
	MRI	Normal	0.1263	0.0368 ~ 0.2157	0.0057
	25-OH-VitD levels				
		Insufficiency	0.039	−0.0444 ~ 0.1224	0.3589
		Sufficiency	0.0095	−0.0803 ~ 0.0993	0.836
		No supplementation	−0.0432	−0.1146 ~ 0.0282	0.2352

While in females, those with a shorter epilepsy duration (≤2 years) had lower serum HDL-C levels than those with a longer history (> 2 years). Serum HDL-C levels were significantly higher in early childhood, middle childhood, and adolescence compared to infancy (β = 0.4298, *p* < 0.0001; β = 0.5049, *p* < 0.0001; β = 0.4326, *p* < 0.0001). HDL-C levels were also significantly lower receiving NEIASMs therapy than in those receiving EIASMs therapy (β = −0.1703, *p* < 0.0001). Interestingly, children with normal MRI results showed higher HDL-C levels than those with abnormal findings (β = 0.1263, *p* = 0.0057).

## 4 Discussion

ASMs are essential for treating epilepsy. Children and adults with epilepsy usually require long-term ASMs therapy, which in some cases may continue for their entire lives (24). It is important to recognize that ASMs may cause various metabolic changes, including alterations in blood lipids (6). Indeed, the long-term adverse reactions of these medications can often be significant, and for many epilepsy patients, these metabolic side effects may become the primary concern (25).

One of the key findings of this study is that serum HDL-C levels in children with epilepsy, regardless of ASMs treatment, are markedly lower than those in healthy children (Figure 3). Serum HDL-C serves as a strong predictor of atherosclerosis risk (26, 27) and is essential for maintaining cholesterol balance between arteries and organs. It is inversely related to the development of coronary heart disease and possesses anti-atherosclerotic properties (28, 29). It is important to note that cardiovascular risk factors, such as lipid abnormalities observed in childhood, may persist into adulthood (30).

There are indeed few reports on the serum HDL-C levels and potential cardiovascular risk factors of children with epilepsy treated with ASMs, and these studies have not yielded consistent conclusions (31, 32). Most research has been conducted on patients without a healthy control group or baseline data. In our current study, both the case and the control groups were selected from the same hospital and within a comparable timeframe. The inclusion criteria comprised individuals diagnosed with epilepsy who had not yet begun treatment with ASMs. To the best of our knowledge, no studies have simultaneously included all three of these subject groups to date.

It is important to reiterate that children who have recently been diagnosed with epilepsy and have not yet begun treatment with ASMs exhibited significantly lower serum HDL-C concentrations compared to healthy children. This means that children with epilepsy may face a potential risk of cardiovascular-related diseases compared to the general population. Interestingly, while some studies are consistent with our results (33, 34), others present conflicting results (35). For example, Verrotti et al. (36) found that serum HDL-C levels in children with epilepsy receiving ASMs treatment were significantly higher than those in the healthy control group, although the cases in that study were adolescents aged 12 and older. In our current study, age subgroup analysis revealed that serum HDL-C levels were notably higher in school-age children compared to younger children. Conversely, Brämswig et al. (37) reported that serum HDL-C levels in healthy volunteers did not change after taking ASMs. Thus, it remains unclear whether the disease itself contributes to the development of low serum HDL-C levels.

Another significant finding of this study is the potential to correct low serum HDL-C levels through ASMs treatment (Figure 4). While previous reports have documented changes in serum HDL-C levels in patients with epilepsy following ASM treatment, there has yet to be an examination of the relationship between epilepsy itself and serum HDL-C levels. Consequently, we particularly focused on the serum HDL-C levels of newly diagnosed patients who had not yet begun ASMs treatment, which provided us with the baseline values.

Surprisingly, the serum HDL-C levels in those children with epilepsy after ASMs treatment was significantly higher than the baseline level (Figure 2). This indicates that ASMs treatment increased serum HDL-C concentrations at the time of diagnosis, although they had not yet reached the levels seen in healthy children. Similarly, Sonmez et al. (10) found that serum HDL-C levels in patients with epilepsy were significantly higher after ASMs treatment compared to prior levels. Therefore, ASMs treatment may help lower the potential risk of CVD in those pediatric patients. It should also be noted that while epilepsy itself is closely associated with low serum HDL-C levels, the causal relationship between the two remains undetermined yet.

There are several aspects that warrant further discussion concerning the impact of ASMs treatment on serum HDL-C levels. Firstly, we observed that the serum HDL-C levels in children with epilepsy receiving EIASMs treatment were significantly higher than those in patients taking NEIASMs treatment, regardless of sex (Figure 4; Table 5). This finding aligns with the study conducted by Yamamoto et al. (38). Interestingly, additional studies have also examined lipid level changes in patients switching from EIASMs to NEIASMs, such as LEV, providing an unique opportunity to analyze the differences in the effects of EIASMs and NEIASMs on serum HDL-C levels within the same individual (39).

Secondly, we conducted a detailed analysis of the effects of ASMs treatment alone on serum HDL-C levels in children with epilepsy. Impressively, the serum HDL-C levels in children receiving OXC monotherapy were significantly higher than those receiving other monotherapies and even reached the levels seen in healthy children (Figure 4). However, the significance disappeared between OXC-monotherapy and other ASM-monotherapy after multivariate adjustment analysis (Table 5). Indeed, there was very limited research evaluating the effect of the OXC treatment on blood lipid levels in children with epilepsy, and no consistent conclusion has been reached to date (31, 40, 41). For example, Franzoni et al. noted a significant decrease in serum HDL-C levels among children with epilepsy (*n* = 28) after 3 months of the OXC monotherapy (40). However, this study could not eliminate the potential influence of the short treatment time and small sample size. Therefore, further research is warranted to confirm the effects of OXC monotherapy on blood lipid levels in children with epilepsy over a longer follow-up period.

Compared with healthy children, the serum HDL-C levels were significantly lower in children with epilepsy taking VPA monotherapy, with no noteworthy difference compared to baseline HDL-C levels before treatment. Furthermore, the data clearly indicated that the median serum HDL-C level in these patients was the lowest observed. Similar findings have been reported (38). Intriguingly, our early meta-analysis suggested that VPA therapy led to a reduction in of total cholesterol (TC) and LDL-C levels (42), while showing no effect on HDL-C levels. However, Franzoni et al. did not identify any significant changes in lipid abnormalities (35). Consequently, the specific effects of VPA on lipid profiles in clinical settings remain difficult to clarify and warrant further investigation.

We found that monotherapy with LEV significantly improved the baseline serum HDL-C levels; however, these levels remained significantly lower than those in children receiving OXC monotherapy. Interestingly, the available evidence suggested that LEV treatment did not affect blood lipid levels or cardiovascular risk factors (43). In addition, there were exceptions regarding the impact of ASMs treatment on HDL-C levels, such as LTG and LAC having no significant effect on HDL-C levels, which is consistent with the findings of Mintzer et al. (44).

In addition, we also observed a weak negative correlation between serum vitamin D and HDL-C levels. Notably, children with vitamin D insufficiency exhibited higher HDL-C levels compared to those with sufficient vitamin D levels. However, the serum HDL-C levels among patients who received vitamin D supplementation were markedly elevated in comparison to those of patients without such supplementation. These results appear to be contradictory, suggesting that the underlying correlations merit further investigation and exploration.

One more question warrants to be further discussed. Generally, serum HDL-C levels below a certain threshold are considered to be unfavorable. According to the reference standards for dyslipidemia in children and adolescents (20), levels <1 mmol/L (40 mg/dL) are classified as “low,” a characteristic of dyslipidemia. In our study, it is evident that, for children with epilepsy-regardless of whether they received ASMs treatment, the proportion of individuals below this level was higher than that of healthy controls. These findings indicate that the CVD risk for children with epilepsy may differ from that of their healthy peers and could be potentially elevated.

The strength of the present study lies in its large sample size, which consisted of 1,002 children diagnosed with epilepsy. Indeed, these were measurements taken over time for the same patient, who may also have changes in all other variables over time (age, body weight, vitamin D dosage, and medication use, etc.), then this constituted a new record, which resulted in the sample size finally increased (*n* = 1884). This study was the largest investigation to date into serum HDL-C levels in children with epilepsy and represented the most extensive clinical study comparing serum HDL-C levels between healthy children and those with epilepsy. Another notable advantage of this study was inclusion of subjects from various groups: healthy children, recently diagnosed epilepsy patients who had not yet begun ASMs treatment, and children who had been on ASMs for varying durations. All subjects were included from the same hospital within a specific timeframe, allowing us to effectively minimize the impact of geographic location and dietary habits when comparing serum HDL-C levels variations among the three groups. Furthermore, this is the first study to evaluate the association between serum vitamin D levels and serum HDL-C levels in both children with epilepsy and healthy children, thereby enhancing our understanding of the CVD risk in this pediatric population.

While this study provides valuable insights, it is subject to several limitations due to its retrospective design. First, the small sample size of children with epilepsy who met criteria for baseline serum HDL-C concentration (*n* = 98) and healthy children (*n* = 127) was a key limitation, the *post hoc* power value between the two groups was only 0.43, which mean the sample size was insufficient to compare the primary outcome, indicating that we need to increase the sample size to further clarify the issue. Second, there was limited data on other ASM monotherapy treatments. Third, CVD was linked to the serum TG, TC, HDL-C, and low-density lipoprotein cholesterol (LDL-C), yet the reasons for these associations are still not fully understood. Although increasing the serum HDL-C level may reduce the likelihood of developing CVD, our retrospective study could not provide additional data, like serum LDL-C concentration, which restricts our ability to thoroughly discuss this novel finding. Fourth, detailed information such as dietary conditions and the supplementation course of vitamin D was not fully collected. Therefore, the correlation between vitamin D and HDL-C has not been thoroughly discussed.

In conclusion, this retrospective study revealed that: (1) regardless of ASMs therapy, children with epilepsy had significantly lower serum HDL-C levels were compared to healthy children, suggesting a potential risk of CVD in this population; (2) treatment with ASMs resulted in a significant increase in serum HDL-C levels, with OXC monotherapy raising these levels to those comparable to healthy individuals. The serum HDL-C levels in untreated children with epilepsy were significantly lower than after treatment, indicating that ASMs therapy unexpectedly improved the low serum HDL-C status associated with epilepsy, which could potentially reduce the risk of CVD in children with epilepsy while managing seizures and enhancing the overall benefits of treatment; (3) supplementation with vitamin D seems to be associated with an increase in serum HDL-C levels. Therefore, the lower serum HDL-C levels in children with epilepsy compared to healthy children, as well as the increase in serum HDL-C levels due to ASMs treatment, should draw attention and warrant further investigation. More research is also needed to confirm these findings and to explore the underlying mechanisms.

## Data Availability

The original contributions presented in the study are included in the article/supplementary material, further inquiries can be directed to the corresponding authors.
